# A pilot study evaluating stress factors during and after the COVID-19 pandemic in Viennese families who have the suspicion of child maltreatment or abuse

**DOI:** 10.1007/s00508-024-02371-z

**Published:** 2024-05-10

**Authors:** Anastasia Pantazidou, Chryssa Grylli, Sophie Klomfar, Eva Mora-Theuer, Johanna Schöggl, Sarah Macura, Laura Schaller, Iulia Pokorny, Susanne Greber-Platzer

**Affiliations:** Forensische Kinder- und Jugenduntersuchungsstelle (FOKUS), Clinical Division for Pediatric Pulmonology, Allergology and Endocrinology, Department for Pediatric & Adolescent Medicine, (Universitätsklinik für Kinder- und Jugendheilkunde), Medical University Vienna, Vienna, Austria

**Keywords:** Child maltreatment, Child abuse, COVID-19 pandemic, Stress factors, Health professional support

## Abstract

The global population was affected by the unprecedented coronavirus COVID-19 pandemic. The impact of the pandemic on children who suffer child maltreatment has not been explored sufficiently. Child abuse is known to increase in stressful circumstances, and therefore potentially during this pandemic.

We aimed to identify and measure the impact of pandemic-related stress in families with a suspicion or confirmed child maltreatment. In addition, other parameters were determined, including resilience factors and family dynamics.

We conducted a pilot study at the Medical University of Vienna, Forensic Examination Centre for Children and Adolescents (FOKUS Safeguarding team). Parents, carers and legal guardians of children who were referred for potential child abuse (study group) participated by completing two questionnaires, one year apart, covering the following periods: pre-COVID, during-COVID and post-COVID. Simultaneously, a control group was devised with patients who presented to the Paediatric Emergency Department with unrelated conditions (other than child maltreatment concerns). The questionnaires addressed psychological stress factors and were completed face-to-face and/or via telephone. A total of 35 carers participated, with almost equal numbers in both intervention and control groups.

Results show that there was statistically significantly higher stress level perception before and during the pandemic period in the study group. Several families in this group commented on the positive effect of support received from health professionals, especially after the pandemic.

## Introduction

The coronavirus pandemic (COVID-19 pandemic) started unexpectedly at the end of 2019, with many countries impacted simultaneously [1]. Data published during and after the pandemic period, as well as towards the endemic period, shows catastrophic numbers of affected individuals and tragic loss of life, amounting to close to 7 million fatal cases. In addition, more recently published data have revealed the concerning symptomatology of Long-COVID, and the long-term outcomes of this new disease entity are yet to be evaluated [2, 3].

Many countries have implemented control measures, including quarantine and lockdowns to reduce disease transmission and consequences, with the aim to contain the spread of the virus [4]. All these measures have imposed additional stress and have generated a considerable amount of psychological pressure on the general population [5].

Child maltreatment is ranking high on the list of potential impacts when there is an increased level of stress. It is well-established that stressful circumstances play an important role in child maltreatment [6, 7]. Child maltreatment may occur as a result of economic hardship, low education level, single parenthood, or having a large number of dependants. Unfortunately, there is not a single factor in isolation that predict child maltreatment. It is common that several risk factors co-exist, with complex interactions between them [8]. Continuous or increasing stress levels in parents have been associated with parenting disengagement [9]. The COVID-19 pandemic was a significant public health challenge that threatened societies all across the world, and it continues to have long-term impacts on family dynamics [10].

Several studies were conducted during the COVID-19 pandemic, but also during the endemic period, almost universally highlighting the negative affect on psychological health on an individual level [11–14]. School closures and other significant changes in everyday lives have had serious psychological effects on adults, as well as the paediatric population. Research shows that children more frequently exhibit symptoms such as inattention, irritability, hyperactivity, anxiety and depression [15]. In addition, their perception of social clues and attainment of resilience has been impacted by the COVID-19 pandemic. Therefore, families with children constitute a population at risk [16]. However, adults have faced disruption in their routines too. The new *norm* during the pandemic was remote working, and many people lost their employment. Subsequently families had to adapt new lifestyle patterns whilst balancing work and personal life [17], with potentially adverse impact on the wellbeing of parents and their children. Resulting parental stress levels could lead to increasing conflicts within the family [18].

The quality of life is significantly reduced in affected family members during periods of crisis relating to health or the economical situation [19]. Research indicates that the impacts of the pandemic in the area of child protection and safety could be detrimental [20].

The aim of this study was to identify and measure the impact of pandemic-related stress in families with a suspicion or confirmed child maltreatment. In addition, other parameters were evaluated, including resilience factors and family dynamics.

## Patients. materials and methods

We conducted a pilot study at the Medical University of Vienna, Department of Pediatrics and Adolescent Medicine, Forensic Examination Centre for Children and Adolescents (FOKUS Safeguarding team). This pilot study aimed to test research protocols, data collection instruments and sample recruitment strategies in preparation for a larger study. It is well known that pilot studies are of importance in the process of a wider research project aiming at identifying potential problem areas and proving the viability of a project idea. The study was approved by the ethics commission of the Medical University Vienna (EK 1365/2020).

We recruited a total of 35 carers during the pandemic period from June 2020 onwards; five participants were lost to follow-up. This study was completed successfully after two years of recruitment and finalized in September 2022.

**Fig. 1:**
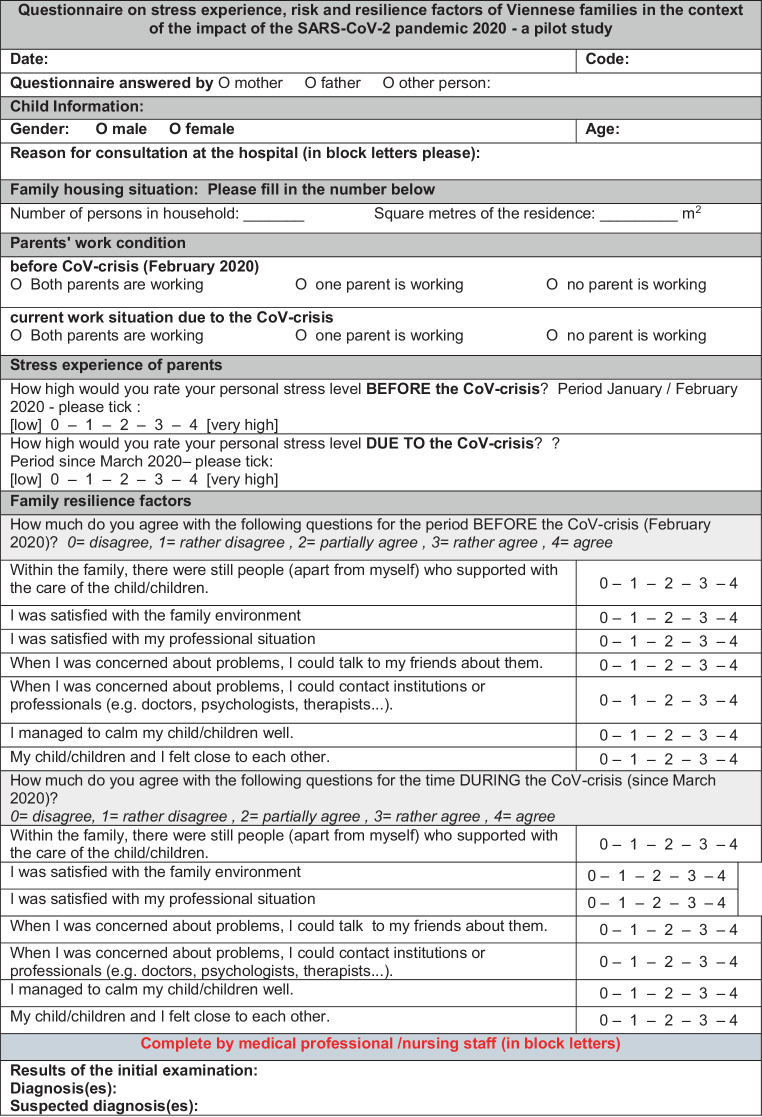
Questionaire 1 (English translation): Questionnaire on stress experience, risk and resilience factors of Viennese families in the context of the impact of the SARS-CoV‑2 pandemic 2020—a pilot study Version 3.0 from 25.05.2020

A survey of the stress factors, as well as the risk and resilience factors, in Viennese families in the context of the effects of the COVID-19 pandemic in connection with the suspicion of child welfare endangerment was carried out in an anonymised manner. Legal guardians of children who were referred to the Forensic Examination Centre for Children and Adolescents (FOKUS Safeguarding team) were consented to participate in this study by completing an anonymised questionnaire (see Fig. 1**Questionnaire 1**) on psychosocial stress factors. The participating families (study group) were referred to the FOKUS Safeguarding team for suspected child physical abuse, sexual abuse and/or neglect. The usual route of referral involved the pediatric emergency unit of the outpatient clinic at the Department of Paediatrics and Adolescent Medicine. After explaining the study and obtaining consent, the questionnaire was filled in by the accompanying person with custody rights directly on site. The family was given the opportunity to have the guidance and help of the team at the Forensic Examination Centre for Children and Adolescents if questions were unclear to them.

In addition, a control group of carers was also randomly enrolled by the FOKUS Safeguarding team as a convenience sample. The families and children who constituted the control group were attending the pediatric emergency unit of the outpatient clinic at the Department of Paediatrics and Adolescent Medicine for reasons other than suspected child maltreatment. In order to be eligible to be recruited into the control group, the corresponding child had to have no chronic co-morbidities. They completed the same questionnaires and the same processes were followed for both the study and the control group throughout.

The interviews were conducted by healthcare professionals, comprising Medical Doctors and Allied Health professionals including psychologists, who recruited the carers, explained the purpose of the study and obtained consent. The questionnaires were conducted as guided interviews.

**Fig. 2:**
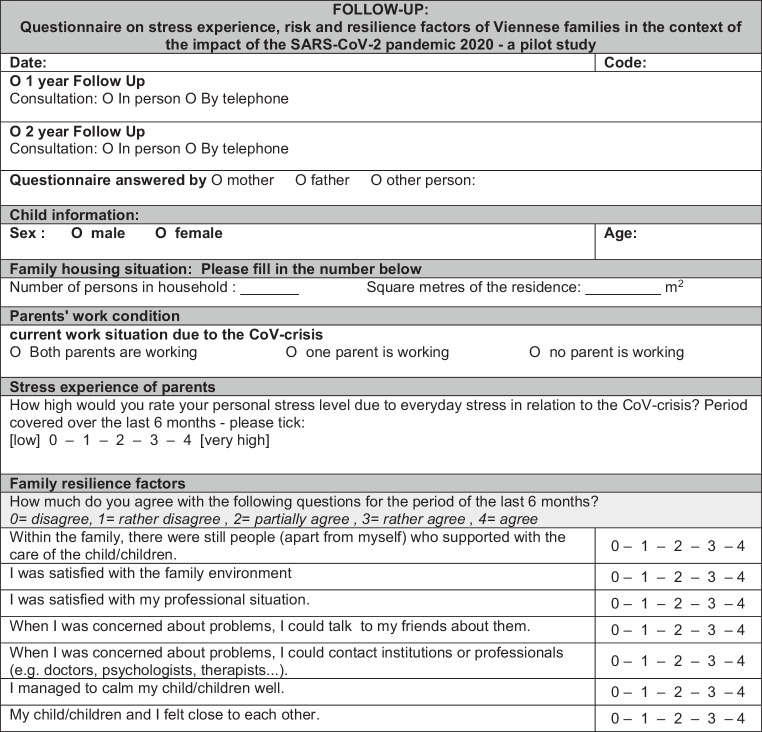
Questionaire 2 (English translation): Questionnaire on stress experience, risk and resilience factors of Viennese families in the context of the impact of the SARS-CoV‑2 pandemic 2020—a pilot study Version 3.0 from 25.05.2020

A year later, a second questionnaire (see Fig. 2*Questionnaire 2*) was completed with the guidance of the same team of healthcare and allied health professionals. At that stage the interviews were conducted over the phone, after reaffirmation of consent. Identified stress factors were evaluated, and if new concerns were identified a second appointment was arranged in addition to the telephone follow-up. This was part of the agreement with families at recruitment. The new data was evaluated and support/guidance was provided if required.

Fig. 1*Questionnaire 1* and Fig. 2*Questionnaire 2* were designed on individual 5‑stage scales, resulting in a maximum total score of 35 in relation to resilience factors. In the section regarding current employment a distinction was made between three levels, depending on whether both parents worked, one parent worked, or neither parent worked. Lastly, the living situation was captured by using the number of people and the number of square meters in the apartment/living accommodation, from which a ratio of people per square meter is devised.

Both questionnaires took approximately 10 min to complete, using a scale question and answer methodology. The questionnaires consisted of 12 questions with demographic data, and 14 interview questions for Fig. 1*Questionnaire 1* and 7 interview questions for Fig. 2*Questionnaire 2* respectively.

Inclusion criteria for the two groups were:

1. An adult present at the time of the child protection referral who is either a birth parent or a legal guardian of the referred child (Child’s age 0–18 years)
2. Main residence in the city of Vienna
3. Well-founded suspicion of child protection concerns with a clear referral for potential child maltreatment (according to article 54 of the Medical Act)
4. Understanding the nature of the study and signing the consent form

The examination of a migration background of the families as a possible factor was not included in this study. In its “EUROPEAN STATUS REPORT ON PREVENTING CHILD MALTREATMENT”, the WHO describes child endangerment as a social problem that exists in all countries [21].

Parents of children with chronic diseases were included in both the study population and the control group. Since chronically ill children have an increased risk of becoming victims, their inclusion is vital [22].

The statistical analysis of the data was performed using IBM SPSS Version 26 (IBM Ltd, Armoni, NY, USA) [23] and Prism OS Catalina Version 7.0 (GraphPad). Groups were compared by using Chi square and Mann Whitney U tests; a *p*-value of < 0.05 was considered statistically significant.

## Results

Out of total of 35 carers who were recruited, five could not be contacted for the follow-up interview arranged a year later. Of those five carers, four were part of the control group and one was part of the study group. The final cohort comprised a total of 17 carers in the control group and 13 in the study group.

Table 1 shows the demographic data of the two groups (study and control group with some comparison). The statistical comparisons showed that there was no significant difference between the groups with regards to the child’s age, the number of persons living in the same household and the living space. However, the average available living space was considerably lower in the study group than in the control group (63.5 vs 86.0 m^2^). It was also notable that the median age was higher in the study group than in the control group (5.5 years vs 3.0 years).

**Table 1:** Baseline demographic data in the study and control group

	Child’s age (years)	*p*-value	No. of persons in household	*p*-value	Living space (m^2^)	*p*-value
Study Group	5.5 (IQR 2.5–8.5)	0.33	3.0 (IQR 2.0–3.3)	0.058	63.5 (IQR 55.8–77.0)	0.36
Control Group	3.0 (IQR 0–10.5)		4.0 (IQR 3.0–4.0)		86.0 (IQR 58.0–114.0)	

Mann-Whitney U test (two-tailed)

**Fig. 3:**
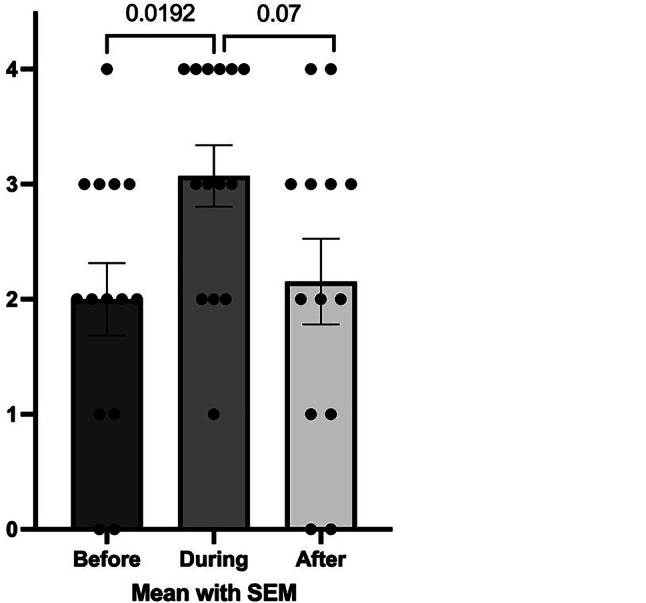
Comparison of stress levels during different periods in the study group

**Fig. 4:**
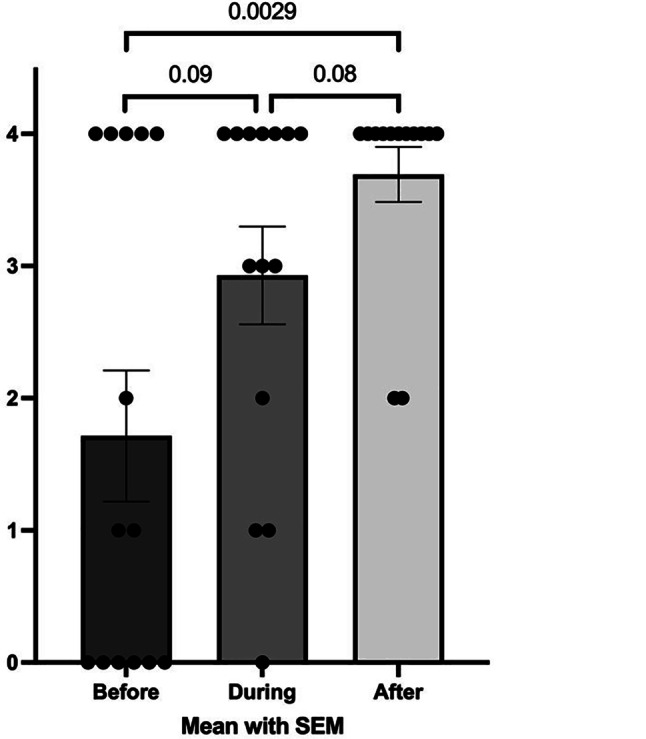
Comparison of the approachability of the health care professionals during different periods in the study group

Table 2 summarises the results of the Questionnaires 1 and 2 in the two separate groups, the study and the control group. Each column represents a specific time period (pre-COVID, during COVID and post-COVID). Statistical comparisons were made within-group across time. As also graphically shown in Fig. 3, in the study group the stress perception increased significantly during COVID, compared to pre-COVID. Also, there was a trend for a decrease of the same variable post-COVID, compared with during COVID. A similar trend was observed in the control group, but this was not statistically significant (Table 2). Figure 4 shows that in the study group there was a significant increase in feeling that they could approach healthcare professionals for help over the three time periods analysed, while this was not observed in the control group.

**Table 2:** Results of Questionnaires 1 and 2 in the study and the control group with comparison between different time periods (pre-/during/post-COVID). Data shown are numbers (people working), medians and interquartile ranges

	Before	During	Before vs. During	After	During vs. After
			*p*-value		*p*-value
**Study group**	*n* = 14	*n* = 14	n/a	*n* = 13	n/a
*Number of people working*					
None	2 (14.3%)	2 (14.3%)	> 0.99^a^	3 (23.1%)	0.45^a^
One	8 (57.1%)	8 (57.1%)		6 (46.2%)	
Both	4 (28.6%)	4 (28.6%)		4 (30.8%)	
*Personal stress load*	2.0 (1.0–3.0)	3.0 (2.0–4.0)	**0.019**	2.0 (1.0–3.0)	0.07
*Childcare support*	2.0 (0.8–4.0)	2.0 (0–3.3)	0.80	2.0 (0–4.0)	0.64
*Household environment*	4.0 (2.0–4.0)	3.0 (2.0–4.0)	0.67	4.0 (3.0–4.0)	0.34
*Employment*	3.5 (2.0–4.0)	3.5 (1.8–4.0)	0.88	3.0 (1.5–4.0)	0.81
*Friendships*	3.5 (1.8–4.0)	3.0 (1.5–4.0)	0.83	4.0 (2.0–4.0)	0.51
*Specialists involved* ^b^	1.0 (0–4.0)	3.5 (1.8–4.0)	0.09	4.0 (4.0–4.0)	0.08
*Ability to calm the children*	4.0 (4.0–4.0)	4.0 (2.8–4.0)	0.26	4.0 (3.5–4.0)	0.61
*Being close to the children*	4.0 (4.0–4.0)	4.0 (4.0–4.0)	> 0.99	4.0 (4.0–4.0)	0.87
**Control group**	*n* = 21	*n* = 21	n/a	*n* = 17	n/a
*Number. of people working*					
None	2 (9.5%)	5 (23.8%)	0.46^a^	2 (11.8%)	0.62^a^
One	12 (57.1%)	10 (47.6%)		10 (58.8%)	
Both	7 (33.3%)	6 (28.6%)		5 (29.4%)	
*Personal stress load*	2.0 (1.0–3.0)	3.0 (2.0–4.0)	**0.045**	2.0 (1.5–3.0)	0.06
*Childcare support*	2.0 (1.0–4.0)	2.0 (0.5–3.5)	0.90	2.0 (0–4.0)	0.78
*Household environment*	4.0 (2.0–4.0)	3.0 (2.0–4.0)	0.91	4.0 (3.0–4.0)	0.32
*Employment*	3.0 (2.0–4.0)	2.0 (0.5–3.0)	0.08	3.0 (2.0–4.0)	0.15
*Friendships*	3.0 (2.0–4.0)	3.0 (2.0–4.0)	0.82	4.0 (4.0–4.0)	0.05
*Specialists involved* ^b^	2.0 (0.5–4.0)	2.0 (1.0–4.0)	0.95	3.0 (2.0–4.0)	0.28
*Ability to calm the children*	4.0 (2.5–4.0)	4.0 (2.5–4.0)	0.87	3.0 (2.0–4.0)	0.71
*Being close to the children*	4.0 (4.0–4.0)	4.0 (3.5–4.0)	0.95	4.0 (3.5–4.0)	> 0.99

^a^Chi Square tests (remaining tests: Mann Whitney *U*)

^b^Note before vs. after: *p* = 0.0029—see graph below

Lastly, it is important to mention that in both study and control groups, the carer-child relationship improved after the COVID-19 pandemic. There is valid literature showing that the stress factors during a pandemic or a disastrous period increase and have a negative impact on the relationships as well as the family dynamics.

Table 3 shows a comparison of the study group and the control group during the different study periods. Only two of the comparisons were statistically significant. Firstly, in the pre-COVID period, carers in the study group had a perceived greater ability to calm their children than the carers in the control group. Secondly, in the post-COVID period, carers in the study group had a greater feeling that they could approach healthcare professionals for help than the carers in the control group.

**Table 3:** Data comparison of the study and control groups during different time periods

	Personal stress load	*p*-value	Childcare support	*p*-value	Household environment	*p*-value	Employment	*p*-value	Friendships	*p*-value	Specialists involved	*p*-value	Ability to calm the children	*p*-value	Being close to the children	*p*-value
*Before*																
SG Before	2.0 (1.0–3.0)	0.81	2.0 (0.8–4.0)	0.95	4.0 (2.0–4.0)	0.84	3.5 (2.0–4.0)	0.68	3.5 (1.8–4.0)	0.99	1.0 (0–4.0)	0.46	4.0 (4.0–4.0)	**0.034**	4.0 (4.0–4.0)	0.51
CG Before	2.0 (1.0–3.0)		2.0 (1.0–4.0)		4.0 (2.0–4.0)		3.0 (2.0–4.0)		3.0 (2.0–4.0)		2.0 (0.5–4.0)		4.0 (2.5–4.0)		4.0 (4.0–4.0)	
*During*																
SG During	3.0 (2.0–4.0)	0.49	2.0 (0–3.3)	0.91	3.0 (2.0–4.0)	0.94	3.5 (1.8–4.0)	0.15	3.0 (1.5–4.0)	0.96	3.5 (1.8–4.0)	0.14	4.0 (2.8–4.0)	0.42	4.0 (4.0–4.0)	0.60
CG During	3.0 (2.0–4.0)		2.0 (0.5–3.5)		3.0 (2.0–4.0)		2.0 (0.5–3.0)		3.0 (2.0–4.0)		2.0 (1.0–4.0)		4.0 (2.5–4.0)		4.0 (3.5–4.0)	
*After*																
SG After	2.0 (1.0–3.0)	0.94	2.0 (0–4.0)	0.93	4.0 (3.0–4.0)	0.94	3.0 (1.5–4.0)	0.98	4.0 (2.0–4.0)	0.22	4.0 (4.0–4.0)	**0.015**	4.0 (3.5–4.0)	0.06	4.0 (4.0–4.0)	0.34
CG After	2.0 (1.5–3.0)		2.0 (0–4.0)		4.0 (3.0–4.0)		3.0 (2.0–4.0)		4.0 (4.0–4.0)		3.0 (2.0–4.0)		3.0 (2.0–4.0)		4.0 (3.5–4.0)	

Abbreviations: *CG* control group; *SG* study group

## Discussion

In this study we tried to identify the impact of stress in families during the COVID-19 pandemic in a specific group of children, who had a suspicion of and potentially suffered child maltreatment. During that period, most families regularly faced significant challenges in their everyday lives. The pandemic has been challenging for many families, and keeping children engaged and safe was a difficult task to accomplish for some [24].

We found that the average available living space was considerably lower in the study group than in the control group. Interestingly, the previous literature shows that environmental factors, including living environment, is relevant to child maltreatment cases. Crowded home settings have a negative impact on child development and wellbeing, including their psychosocial progress [25, 26].

Comparisons between the study and the control groups at different time periods (pre-COVID, during COVID and after COVID) revealed subtle differences between the two. Firstly, during the pre-COVID period carers in the study group estimated their ability to calm their children higher than those in the control group. Secondly, after COVID carers in the study group found that it was easier to approach health care professionals than did carers in the control group. Also, we found in the study group that carers’ perception of the accessibility of healthcare professional increased over the three time periods studied. Those observations are interesting, but it has to be considered that carers in the study group may deliberately have provided ‘desirable’ answers, rather than providing answers that reflect the actual situation.

In the study group there was a statistically significant increase in stress perception during COVID, compared with the pre-COVID period. A similar trend was observed in the control group, but this did not reach statistical significance. One should never underestimate the impact of unforeseen circumstances on vulnerable families in comparison to families with balanced dynamics [27, 28]. However, adapting coping strategies can serve protectively to individuals who experience stress [29].

Some limitations of this study have to be mentioned. Firstly, this is a pilot study with a limited number of participants and refers to a specific, tertiary setting. It is unfortunate that a small number of participants were lost to follow up. Given that this was a pilot study and only included a comparatively small number of statistical comparisons, we chose not to correct for multiple statistical tests (such as using Bonferroni correction), which may have introduced bias. To explore the issues regarding parental stress factors and the association with child maltreatment in a pandemic setting in more detail, further studies with a larger number of participants are required. Secondly, the data collected reflects the parental views of the child’s wellbeing; additional evaluation assessing the children’s views may uncover additional issues. Finally, we only determined a limited number of factors and outcomes.

Despite those limitations, our aim with this study was to raise awareness and explore risk factors within the family environment in vulnerable situations. Also, we would like to highlight the importance of specifically considering at-risk families for future policy development and improvement of services. Child maltreatment, regardless of a pandemic situation, will always be the responsibility of the government bodies, but also of individuals involved in the child’s care [30]. Much like other services, child protection services experienced significant challenges during the pandemic period, and perhaps received less attention than it should have.

## Conclusion

Without a doubt the COVID-19 pandemic has negatively affected many aspects of children’s lives. With this pilot study we tried to raise awareness of the importance of maintaining child protection services to keep the children safe [3, 8].

Interestingly our data show that families respond diversely to stress levels and many families have dealt with significant challenges during the pandemic period. Our data highlights the importance of child protection services working continuously without disruption, despite difficult circumstances. Vulnerable families have benefited from the presence and help received from Healthcare and Allied Health professionals and other relevant organisations.

Lastly, finding innovative solutions to support child protection services in situations such as the COVID-19 pandemic is vital. Strengthening partnerships and recognizing limitations will help to move forward with ensuring safe environments to enhance child development and limit child maltreatment.

## Abbreviations

COVID-19: Coronavirus disease of year 2019
FOKUS: Forensic Examination Centre for Children and Adolescents “Forensische Kinder- und Jugenduntersuchungsstelle”
IQR: Interquartile range
